# Dual role of the chromatin-binding factor PHF13 in the pre- and post-integration phases of HIV-1 replication

**DOI:** 10.1098/rsob.170115

**Published:** 2017-10-11

**Authors:** Stephan Hofmann, Sandra Dehn, Ramona Businger, Sebastian Bolduan, Martha Schneider, Zeger Debyser, Ruth Brack-Werner, Michael Schindler

**Affiliations:** 1 Helmholtz Zentrum München, German Research Center for Environmental Health, Institute of Virology, Neuherberg, Germany; 2 Institute of Medical Virology and Epidemiology of Viral Diseases, University Hospital Tübingen, Tübingen, Germany; 3 Molecular Virology and Gene Therapy, KU Leuven, Leuven, Belgium

**Keywords:** HIV-1, PHF13, integration, Vpr, virus restriction factor

## Abstract

Viruses interact with multiple host cell factors. Some of these are required to promote viral propagation, others have roles in inhibiting infection. Here, we delineate the function of the cellular factor PHF13 (or SPOC1), a putative HIV-1 restriction factor. Early in the HIV-1 replication cycle PHF13 increased the number of integrated proviral copies and the number of infected cells. However, after HIV-1 integration, high levels of PHF13 suppressed viral gene expression. The antiviral activity of PHF13 is counteracted by the viral accessory protein Vpr, which mediates PHF13 degradation. Altogether, the transcriptional master regulator and chromatin binding protein PHF13 does not have purely repressive effects on HIV-1 replication, but also promotes viral integration. By the functional characterization of the dual role of PHF13 during the HIV-1 replication cycle, we reveal a surprising and intricate mechanism through which HIV-1 might regulate the switch from integration to viral gene expression. Furthermore, we identify PHF13 as a cellular target specifically degraded by HIV-1 Vpr.

## 1. Background

Viruses hijack and reprogram the host cell machinery in order to achieve optimal viral replication and multiplication. For a variety of viral infections, including HIV-1, large efforts have been undertaken to identify cellular genes which are beneficial or necessary for productive infection, so-called host dependency factors [1–4]. On the other hand, host cells have evolved potent antiviral strategies in order to suppress and restrict virus infection and production, which are designated restriction factors [5]. Thus, knowledge of cellular factors that are beneficial as well as inhibitory for viral infections is of fundamental importance to tailor novel therapeutics and antiviral strategies.

Host cell factors are often manipulated by HIV-1 accessory proteins (i.e. Nef, Vpu, Vif and Vpr) [6]. These are mostly dispensable for HIV-1 production in cell culture but important for the maintenance of high viral loads and progression to AIDS *in vivo*. One important function of HIV-1 accessory proteins is to achieve evasion from the host's immune response for instance by downmodulation of cell surface immune receptors or through counteraction of cellular antiviral restriction factors. In recent years research efforts have delineated major functions of Vpu, Vif and Nef [6]. By contrast, Vpr remains one of the most enigmatic HIV-1 accessory proteins [7].

Vpr is a 12.7 kDa small protein consisting of three amphipathic helices with the capacity to form oligomers [7]. Vpr enhances HIV-1 replication in human lymphoid tissue (HLT) [8], non-activated CD4^+^ T cells [9], macrophages [10] and some immortalized T cell lines [11]. This enhancement could be related to its ability to increase nuclear import of the HIV-1 pre-integration complex, activation of various transcription factors including NFAT (nuclear factor of activated T cells) and a direct stimulation of HIV-1 LTR transactivation [7,12]. One of the best-investigated Vpr phenotypes is its ability to induce a G_2_ cell cycle arrest, which is related to the association of Vpr with a larger ubiquitin ligase complex composed of VPRBP (Vpr binding protein or DCAF), DNA damage-binding protein 1 (DDB1) and the ubiquitin ligase cullin 4A (CUL4A) [13]. However, the physiological relevance of Vpr-mediated G_2_ arrest remains unclear [7], although it was proposed to be relevant for HIV-1 evasion from immune sensing [14] and depletion of T_regs_ in the context of CCR5-tropic HIV-1 infection [15].

PHF13 (PHD finger protein 13 or SPOC1; survival-time associated PHD finger in ovarian cancer 1) was originally identified as a potentially oncogenic cellular factor due to the abundance of increased RNA levels in ovarian tumour tissue which was associated with decreased survival probability of cancer patients [16]. It is conserved from zebrafish to humans and involved in a multitude of processes, including regulation of DNA damage response [17,18] and development [19]. PHF13 is 300 amino acids in length migrating at a MW of 43 kDa. It contains a bipartite nuclear localization signal, two PEST domains, a conserved C-terminal plant-homeodomain zinc finger (PHD) through which it binds to chromatin and a conserved N-terminal domain [20]. PHF13 was reported to regulate cell division through the association with chromatin thereby influencing its condensation [20]. In addition, PHF13 is recruited to DNA double-strand break (DSB) repair loci post-induction of DNA damage, implying an important role in the regulation of the DNA damage response [17,18]. Recently, the underlying mechanism of PHF13 targeting to chromatin was elucidated [21]. It directly interacts with H3K4me2/3 DNA and associates with polycomb repressive complex 2 (PRC2) as well as RNA PolII. Thereby it intriguingly up- and downregulates multiple genes involved in transcriptional regulation, DNA binding and chromatin organization, and cell cycle regulation and differentiation [21]. In addition, the PHF13 interactome is enriched for approximately 50 spliceosomal proteins [22]. Altogether, this suggests that PHF13 is a transcriptional co-regulator at H3K4me2/3 that couples transcription with co-transcriptional splicing [21,22].

PHF13 was reported to repress gene expression of adenovirus and the authors speculated that this might be a general host defense mechanism of antiviral restriction, because PHF13 expression was also reduced in lysates of an HIV-1 infected T cell line [23]. We therefore investigated the role of PHF13 during HIV-1 infection and found that the expression of PHF13 is tightly regulated throughout the viral replication cycle. In the first few hours after viral entry PHF13 stimulates HIV-1 integration and its levels are unaffected. However, upon completion of proviral integration, PHF13 is degraded by virion-delivered Vpr, probably due to a repressive effect of PHF13 on HIV-1 gene expression.

Altogether, oppositely what we expected, PHF13 has positive as well as negative effects on viral replication dependent on the stage of HIV-1 infection.

## 2. Material and methods

### 2.1. Cell culture, plasmids and proviral constructs

293T were cultivated in DMEM supplemented with 10% FCS (Gibco), Pen/Strep (120 µg ml^−1^) and 350 µg ml^−1^ l-glutamine. U2OS cells and PHF13-inducible U2OS-C5 cells [20] were both kindly contributed by Hans Will and cultured in DMEM with standard supplements and additionally with 1% HEPES buffer solution (Gibco). Jurkat-TAg (kindly provided by O. Fackler), SupT1 and J-Lat cells (both from the NIH AIDS Reagent Program) were grown in RPMI1640 supplemented with 10% FCS, Pen/Strep, l-glutamine and 1% sodium pyruvate (Gibco). For the generation of primary human macrophages and CD4^+^ T cells, we isolated peripheral blood mononuclear cells (PBMCs) from buffy coat received from the German Red Cross by Ficoll gradient centrifugation. Macrophages and CD4^+^ T cells were generated and cultured as described elsewhere [24]. All cells were grown in a 5% CO_2_ atmosphere at 37°C. Vpr pCG-expression plasmids and the pCG-IRES-GFP have been described previously [9,25]. Similarly, PHF13 was amplified from cDNA (kindly contributed by Hans Will) and ligated into the pCG-IRES-mTagBFP vector [26]. All PCR-derived inserts were sequenced to confirm their nucleotide identity. HIV-1 NL4–3 WT, ΔNef, ΔVpu and ΔVpr proviral constructs and mutants have been described previously [8,9,27]. For some experiments, we used similar HIV-1 NL4–3 variants co-expressing Nef and GFP via an IRES, which is an indicator for LTR transactivation [28,29]. Vpr pWPI lentiviral constructs were kindly contributed by E. Cohen [30].

### 2.2. Generation of HIV-1 stocks and infection experiments

In order to allow CD4-independent infection of target cells and increase infection efficiencies vesicular stomatitis virus glycoprotein (VSVG) pseudotyped HIV-1 stocks were generated by standard calcium phosphate transfection of 293T cells essentially as described previously [9,20,28]. Briefly, 293T cells were cotransfected with the various NL4-3 proviral constructs and the pHIT-G plasmid coding for the envelope protein of the VSVG. Thirty-six hours later supernatants were harvested, cleared by centrifugation and stored at 4°C until infection of target cells. For infection of U2OS or U2OS-C5 cells 3 × 10^5^ cells were seeded in six-well plates and infected one day later with 200 ng p24 HIV-1 viral stocks. If not indicated otherwise, 24 h later cells were washed and replaced with fresh media. Similarly, 2 × 10^6^ SupT1, Jurkat-Tag or primary CD4^+^ T cells were cultivated in 2 ml RPMI in six-well plates and infected with 200 or 400 ng p24 HIV-1 viral stocks. Vpr transcomplementation and infection experiments were done as previously described [9].

### 2.3. Immunoblotting and antibodies

For protein analysis cells were lysed in RIPA buffer (50 mM Tris–HCl pH 7.4, 150 mM NaCl, 2 mM EDTA, 1% NP-40, 0.1% SDS) supplemented with 1× complete protease inhibitor cocktail (Roche). After 10 min on ice, the insoluble debris was pelleted at 10 000*g*/4°C for 10 min. Supernatant was diluted with SDS loading buffer, heated at 95°C for 5 min before loading on a SDS-PAGE. Immunoblotting and SDS-PAGE were performed using standard protocols. Blocking was done by incubation in 10% [w/v] milk powder in TBS-T for 1 h at RT with shaking. Incubation with primary antibody (diluted in 5% [w/v] milk powder in TBS-T) was performed overnight at 4°C with constant inversion. Alternatively, we used the established protocol for PHF13 protein analysis and immunoblotting [20,21]. Primary antibodies used in this study included mouse anti-HIV-p24 (1 : 5000; Abcam), rabbit anti-HIV-Vpr (1 : 2000; kindly provided by Ulrich Schubert [31]), monoclonal rat anti-PHF13 (1 : 50; kindly provided by Elizabeth Kremmer [20]), mouse anti-tubulin (1 : 1000; Sigma) or mouse anti-actin (1 : 1000; Sigma). Secondary Ab conjugated to horseradish peroxidase were anti-rat IgG, anti-rabbit IgG (Jackson Immuno Research) and anti-mouse IgG (Dianova). Immunoblots were visualized by using the Fusion X7 camera system (Peqlab). For LI-COR Odyssey Imaging System based detection we used IRDye 800 CW goat anti-rabbit and goat anti-rat or goat anti-mouse IRDye 680 RD (1 : 15 000; Li-COR Biosciences).

### 2.4. Knockdown of PHF13 in U2OS cells by siRNA

U2OS-C5 cells were transfected with 100 nM PHF13 specific siRNA (5′ UCACCUGUCCUGUGCGAAA 3′) or iBoni control N2 siRNA (riboxx) with Lipofectamine 2000 (Invitrogen) using the standard protocol provided by the manufacturer and as described previously [20].

### 2.5. Inhibitor treatment experiments

Proteasomal inhibitors were used in culture for up to 6 h at 5 µM MG132 (Calbiochem) and 5 µM lactacystin (Sigma). Calpain was inhibited by treatment of cells in culture for 24 h with 50 nM calpain inhibitor 1 (CI1 also called ALLN or MG101; Sigma). GSK3β was inhibited by incubating cells in culture for 6 h with 100 nM insulin (Sigma) or the specific inhibitor SB216763 (Sigma) in increasing amounts (10–100 µM). Neddylation inhibitor MLN4924 was purchased from BostonBiochem and applied in a final concentration of 0.1 to 1 µM. HIV-1 inhibitors were used in the following concentrations: Raltegravir 250 nM (Santa Cruz), Flavopiridol 50 nM, Efavirenz 50 nM, Saquinavir 50 nM (all from the NIH AIDS Reagents Program).

### 2.6. ELISA to assess HIV-1 p24 capsid production

Virus stocks or cell supernatants were lysed with Triton X-100 (Sigma) at 4°C for 12 h. HIV-1 p24 Antigen Capture Assay Kit (ABL Inc.) was used to measure the amount of the capsid protein p24 as recommended by the manufacturer's protocol. Unbound material was removed by several washing steps with PBS. Addition of Peroxidase substrate (KPL) leads to a colour change of the solution, which was stopped by adding 100 µl stop solution. Absorbance was measured at 450 nm in an Infinite M200 (Tecan) and corrected for 650 nm reference wavelength. Absolute p24 amounts were calculated by measuring a sequential dilution and generation of a calibration curve.

### 2.7. Alu-PCR to measure the amount of integrated proviral genomes

Cells were harvested 24 h after infection and chromosomal DNA was extracted with the GeneJET Genomic DNA purification Kit (Thermo). The Alu LTR-based real-time nested PCR is a method to quantify the integrated HIV-1 proviral DNA in infected cells [32]. In the first PCR step, integrated HIV-1 sequences were amplified with outward-facing Alu primer 5′-TCCCAGCTACTGGGGAGGCTGAGG-3′ and HIV-1 specific primer 5′-ATGCCACGTAAGCGAAACTCTGGCTAGCTAGGGAACCCACT-3′. Conditions for PCR were denaturation at 95°C for 10 min, subsequently 15 PCR cycles with 95°C for 10 s, 60°C for 10 s and 72°C for 2 min 50 s. Of note, 2 µl of the products served as template in the second nested PCR step using viral LTR primer 5′-TGCTAGAGATTTTCCACACTGACTAAAAGGG-3′ and 5′-ATGCCACGTAAGCGAAACT-3′. Conditions of the second nested PCR were as follows: 95°C for 10 min, 45 cycles at 95°C for 10 s, 60°C for 5 s, 72°C for 10 s. All PCRs were performed in a Light Cycler LC 480 (Roche). As reference gene we used β-globin, which was quantified with forward primer 5′-ACACAACTGTGTTCACTAGC-3′ and reverse primer 5′-CAACTTCATCCACGTTCACC-3′. Relative proviral copy numbers were quantified using the method of Pfaffl [33,34].

### 2.8. PHF13 overexpression and microporation

PHF13 overexpression was induced by treatment of U2OS-C5 cells for 16–24 h with 1 µg ml^−1^ doxycycline (Sigma). Additionally, human PHF13 was expressed from cells microporated with pCG-PHF13-IRES-mTagBFP vector or the mTagBFP-only vector control. Jurkat-TAg or SupT1 cells were microporated using the Neon (Life Technologies) transfection system and the Jurkat-specific protocol available from the Life Technologies website. Briefly, 1 × 10^6^ cells were washed with PBS, centrifuged at 400*g* for 5 min and the supernatant was discarded. The cell pellet was resuspended in the provided buffer solution containing the DNA and electroporated with three electric pulses (1350 V, 10 ms). Afterwards, cells were transferred in pre-warmed RPMI1640 media without antibiotics and cultivated for 24–48 h at 37°C, 5% CO_2_ to yield optimal levels of protein expression. DNA or siRNA amounts for 1 × 10^6^ cells were 5 µg of plasmid DNA or 100 nM siRNA, respectively.

### 2.9. Software and statistics

For data analysis we used Microsoft Excel or GraphPad Prism 5.0 and 6.0. Densitometric immunoblot analysis was done with the Licor build-in software package. CorelDraw X7 was used for the generation of figures and Microsoft Word as well as EndNote X7 for manuscript writing. Statistical significance was assessed with GraphPad Prism 5.0 and 6.0. The used respective statistical test is indicated in the according figure legends.

## 3. Results

### 3.1. PHF13 levels are reduced upon HIV-1 infection

**Figure 1.**
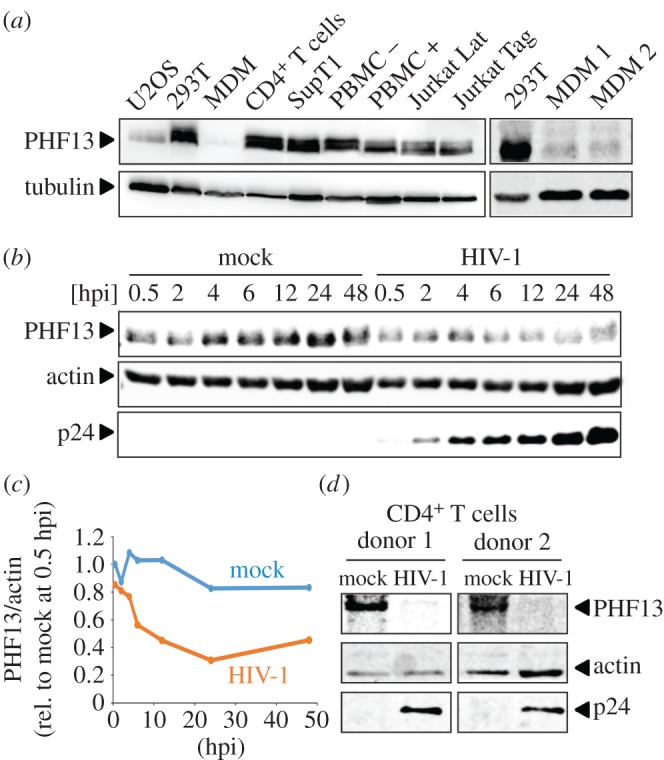
PHF13 is expressed in HIV-1 target cells and its expression is reduced early post-infection. (*a*) Total cellular lysates of the non-infected indicated cell lines and primary cells were subjected to immunoblot for detection of PHF13 (43 kDa) and tubulin as described in the Material and methods section. One representative of at least three independent blots is shown. (*b*) SupT1 cells were infected with 200 ng p24 VSVG pseudotyped HIV-1 NL4-3 or mock infected. Cells were harvested at the indicated time points post infection, total cell extracts were prepared and analysed for expression of PHF13, HIV-1 capsid p24 and actin by immunoblot. Similar results were obtained in two additional biological replicates. (*c*) Densitometric analyses of the data shown in (*b*). Values are normalized to total protein content (actin) by dividing the intensity of PHF13 by the corresponding actin intensity. Protein expression was calculated relative to the mock 0.5 hpi time point which was set to 1. (*d*) Primary CD4^+^ T cells from two different donors were infected with 400 ng p24 VSVG pseudotyped HIV-1 NL4-3 or mock infected. Cells were harvested 48 hpi, total cell extracts were prepared and analysed for expression of PHF13, HIV-1 capsid p24 and actin by immunoblot.

PHF13 represses gene expression of adenovirus and the authors speculated that PHF13 might generally act as a virus restriction factor, including HIV-1 as they observed reduced PHF13 levels in an HIV-1 infected T cell line [23]. We first clarified whether PHF13 is expressed in non-infected cell lines relevant for production and infection of HIV-1 as well as primary target cells (i.e. PBMC, CD4^+^ T cells and macrophages; figure 1*a*). As a reference we used U2OS cells, because previous studies on PHF13 were mainly conducted in this cell line [20]. PHF13 was robustly expressed in 293T cells, which are standard to produce infectious HIV-1 stocks from transfected proviruses and all other cell lines tested. This includes the immortalized T cell lines SupT1 and Jurkat, both being CD4^+^ T cell lines widely used in HIV-1 research, as well as Jurkat latently infected with HIV-1 (Jurkat-Lat). In addition, PHF13 was expressed in unstimulated and PHA-treated PBMC and primary CD4^+^ T cells. Macrophages had substantially lower, albeit detectable PHF13 expression (figure 1*a*). Furthermore, PHF13 levels were dramatically lower in HIV-1-infected T cells in comparison with uninfected controls, suggesting that HIV-1 actively reduces PHF13 expression (electronic supplementary material, figure S1). We next measured the dynamics of PHF13 reduction upon HIV-1 infection. SupT1 CD4^+^ T cells were infected with HIV-1 and aliquots of the infected culture were taken at different time points post infection. These were subjected to immunoblotting for the detection of PHF13, p24 (HIV-1 capsid protein) and actin (figure 1*b*). In comparison with mock-infected SupT1 cells we detected substantial reduction of PHF13 in infected cells already at 4 hpi, which reached a maximum at 24 hpi (figure 1*b* and quantification figure 1*c*). We also detected reduced PHF13 levels in primary HIV-1-infected CD4^+^ T cells (figure 1*d*). From these data we conclude that HIV-1 reduces PHF13 levels early post-infection in a time-dependent manner in virally infected T cells.

### 3.2. HIV-1 Vpr induces reduction of PHF13 steady state expression

HIV-1 has evolved a repertoire of multi-functional accessory proteins, which are required for effective immune evasion and the maintenance of high viral loads [6]. For instance, Vpu counteracts the antiviral restriction factor Tetherin [35,36] and Nef inhibits cell surface expression of MHCI to evade lysis of infected cells by cytotoxic T lymphocytes [37]. In contrast, the cellular target(s) of Vpr are less defined, although Vpr has recently been described to activate the SLX4 complex in order to suppress the innate immune response against HIV-1 [14] and it degrades the DNA repair helicase HLTF [38–40].

**Figure 2.**
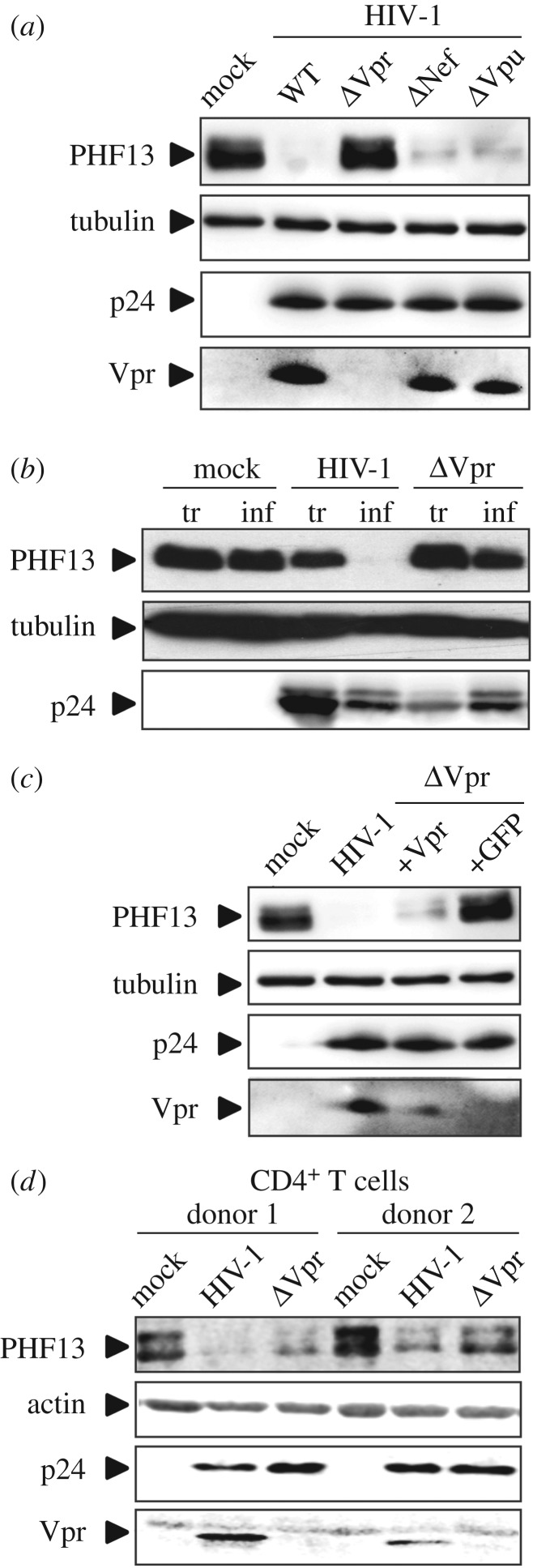
Vpr is the viral determinant responsible for PHF13 reduction. (*a*) SupT1 cells were infected with equal p24 amounts (200 ng) of VSVG pseudotyped HIV-1 NL4-3 IRES-eGFP or variants with inactivating mutations in Vpr, Nef or Vpu. Forty-eight hours post-infection cell lysates were subjected to immunoblot against PHF13, HIV-1 p24, tubulin and Vpr. (*b*) 293T cells were either transfected with equal DNA amounts of the indicated HIV-1 NL4-3 IRES-eGFP constructs or infected with same p24 amounts (100 ng) of the respective VSVG pseudotyped HIV-1 NL4-3 IRES-eGFP virus stocks. Thirty-six hours later cells were analysed by immunoblot for PHF13, HIV-1 p24 and tubulin levels. (*c*) SupT1 cells were infected with 200 ng p24 of VSVG pseudotyped HIV-1 NL4-3 IRES-eGFP or the ΔVpr variant, transcomplemented in the 293T producer cells with Vpr or GFP only. Forty-eight hours later, cells were lysed and subjected to immunoblot for detection of PHF13, HIV-1 p24, tubulin and Vpr. In addition to p24 quantification all transfections or infections presented in (*a*)–(*c*) were analysed by flow cytometry for the % of GFP+ cells. These were similar within experiments and in the range of 50 to 90%. All immunoblots presented in (*a*)–(*c*) were confirmed in at least two additional independent experiments. (*d*) Primary CD4^+^ T cells from two different donors were infected with 400 ng p24 VSVG pseudotyped HIV-1 NL4-3, a variant with inactivated Vpr ORF or mock infected. Cells were harvested 48 hpi, total cell extracts were prepared and analysed for expression of PHF13, HIV-1 capsid p24, actin and Vpr by immunoblot.

We hypothesized that reduction of PHF13 expression might be due to the action of an HIV accessory protein. We hence infected SupT1 cells with HIV-1 NL4-3 variants containing inactivating mutations in the accessory proteins Vpr, Nef and Vpu, and measured PHF13 protein levels (figure 2*a*). WT HIV-1-infected SupT1 cells displayed strongly reduced PHF13 levels, and the same was true for infection with Nef- and Vpu-deficient HIV-1. Strikingly, infection with Vpr-deleted HIV-1 (ΔVpr) resulted in PHF13 levels comparable with mock-infected cells (figure 2*a*).

We next aimed to set up an easy system to analyse Vpr-mediated PHF13 reduction in transfected 293T cells. However, Vpr expression alone was not sufficient to reduce PHF13 protein levels (data not shown). We hence considered that (i) 293T cells might not support Vpr-mediated PHF13 reduction, (ii) additional viral proteins could be necessary to reduce PHF13, and (iii) degradation of PHF13 may occur only in the context of HIV-1 replication and therefore cannot be recapitulated by transfection of HIV-1 proteins. Hence, we either transfected or infected 293T cells with WT HIV-1 and the ΔVpr variant (figure 2*b*). Transfection with full-length HIV-1 resulted in only marginally reduced PHF13 levels, despite transfection efficiencies greater than 90% and high p24 levels. In contrast, infection of 293T cells with VSVG pseudotyped HIV-1 led to a complete loss of PHF13 expression and this phenotype was again clearly attributable to Vpr (figure 2*b*).

Vpr is incorporated into newly synthesized virions and, therefore directly present in infected cells prior to de novo synthesis of viral proteins. To analyse whether virion-delivered Vpr is sufficient for PHF13 reduction we used ΔVpr HIV-1 and transcomplemented Vpr in the producer cell by cotransfection of the pCG-Vpr expression plasmid, similar to our previous experiments [9]. When SupT1 cells were infected with WT HIV-1 or ΔVpr that was transcomplemented with Vpr, reduction of PHF13 was robust (figure 2*c*). In contrast, control cells infected with ΔVpr HIV-1 which was transcomplemented with GFP only showed PHF13 levels similar to mock-infected cells. From this data we conclude that virion-delivered Vpr is sufficient to cause reduction of PHF13 in the infected host cell. Furthermore, we demonstrate the importance of Vpr for reduced PHF13 expression in primary HIV-1-infected CD4^+^ T cells (figure 2*d*).

### 3.3. PHF13 is degraded in a GSK3β and calpain-dependent manner post integration of the proviral HIV-1 genome

**Figure 3.**
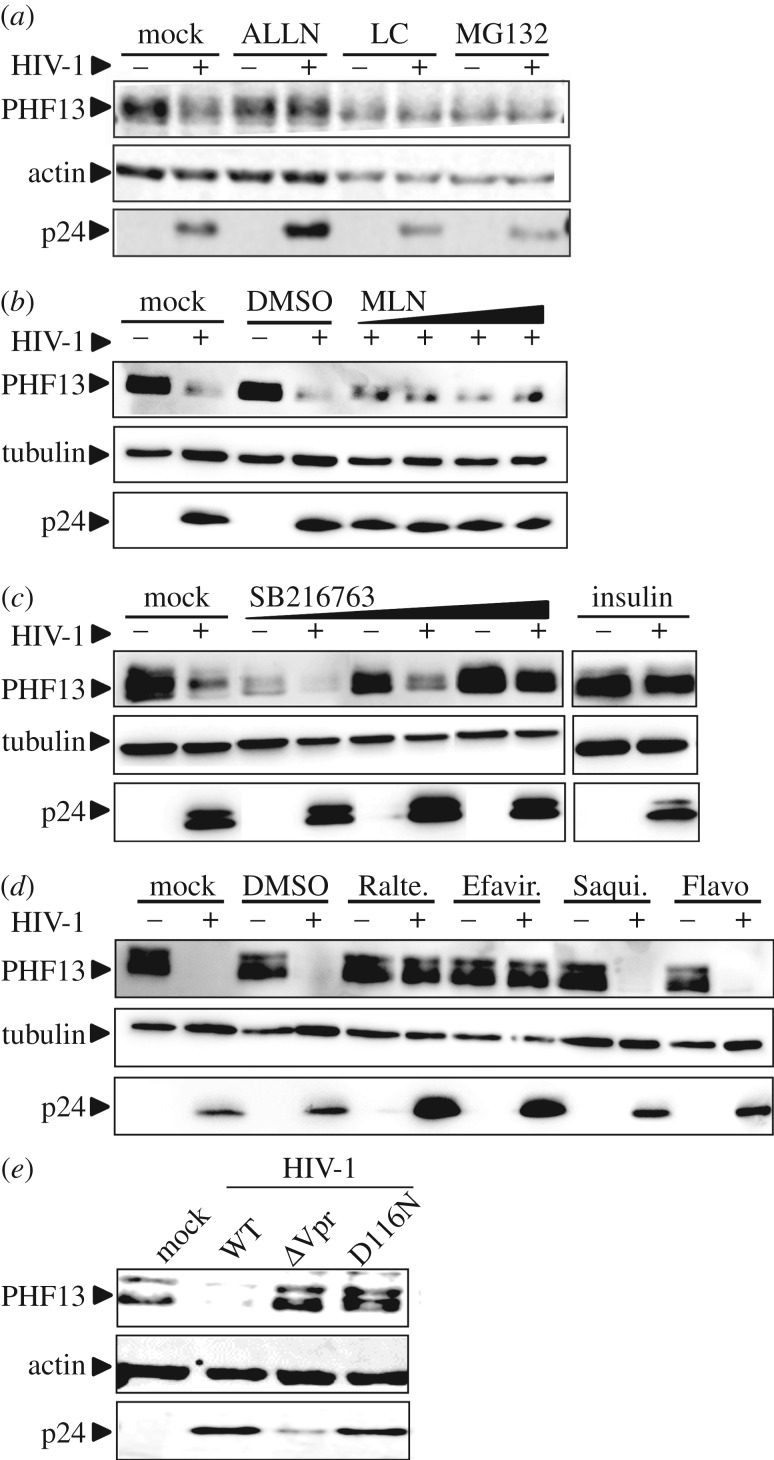
PHF13 is degraded by the proteasome in a GSK3β-dependent manner early post-integration. (*a*) SupT1 cells were infected with equal amounts of VSVG pseudotyped HIV-1 NL4-3 IRES-eGFP. Simultaneously, cells were treated with 50 nM calpain Inhibitor I (ALLN), or at 6 hpi with 5 µM proteasome inhibitors lactacystin (LC) or MG132 for additional 6 h. PHF13, actin and p24 expression were analysed by immunoblotting. The same result was obtained in one additional experiment. (*b*) SupT1 cells were incubated with increasing amounts (0.1; 0.25; 0.5; 1 µM) of MLN4924, DMSO or were mock treated for 3 h. Subsequently cells were infected with 200 ng p24 VSVG pseudotyped HIV-1 NL4-3 IRES-eGFP. Forty-eight hours post-infection lysates were generated and analysed for PHF13, tubulin and p24 expression by immunoblotting. (*c*) SupT1 cells were treated with increasing amounts (10; 40; 100 µM) of GSK3β inhibitor SB216763 or 100 nM insulin for 6 h, followed by infection with 200 ng p24 VSVG pseudotyped HIV-1 NL4-3 IRES-eGFP. Forty-eight hours post-infection cells were analysed by immunoblot for PHF13, tubulin and p24 expression. (*d*) SupT1 cells were infected with 200 ng p24 VSVG pseudotyped HIV-1 NL4-3 IRES-eGFP, simultaneously cells were incubated with different drugs inhibiting various steps of HIV-1 replication. Forty-eight hours post-infection cells were lysed and subjected to immunoblot for the detection of PHF13, tubulin and HIV-1 p24. Raltegravir was used at 250 nM, whereas Efavirenz, Saquinavir and Flavopiridol were used at 50 nM. (*e*) SupT1 cells were infected with 200 ng p24 of VSVG pseudotyped HIV-1 NL4-3, the ΔVpr variant or HIV-1 with mutation D116N, blocking integration. Forty-eight hours later, cells were lysed and subjected to immunoblot for detection of PHF13, HIV-1 p24 and actin. The data presented in (*b*)–(*e*) were all confirmed in at least two additional independent replicates. Furthermore, for (*a*)–(*d*) the % of GFP+ cells was analysed in all experiments to control for equal infection rates. With Raltegravir and Efavirenz the number of infected (GFP+) cells was reduced to background levels.

Generally, the HIV-1 accessory proteins are adaptors which induce the degradation of cellular factors [6]. To test whether proteases or the proteasome are involved in HIV-1-mediated PHF13 degradation, we treated HIV-1-infected SupT1 cells with ALLN, a specific calpain inhibitor, lactacystin and MG132, both inhibitors of the proteasomal degradation machinery [41,42]. This set-up was chosen because MG132 is less specific than lactacystin and additionally inhibits calpains, calcium-dependent, non-lysosomal cysteine proteases [43]. Although MG132 and lactacystin were fairly toxic in SupT1 cells, they clearly stabilized PHF13 expression at a level similar to non-infected cells (figure 3*a*) and this phenotype was confirmed in HIV-1 infected 293T cells (data not shown). Furthermore ALLN prevented PHF13 degradation by HIV-1 (figure 3*a*).

The canonical pathway of Vpr-mediated protein degradation is through association with an E3 ubiquitin ligase complex which might ultimately lead to the degradation of cellular targets [7,13], including HLTF [38–40]. The activity of this complex can be inhibited by the neddylation inhibitor MLN4924 [44]. Treatment of HIV-1-infected SupT1 cells with increasing concentrations of MLN4924 did not result in stabilization of PHF13, but rather showed PHF13 levels in infected cells which were similar to mock or DMSO cells (figure 3*b*). This suggests that neddylation does not play a major role in PHF13 degradation by HIV-1.

Calpains are calcium activated [45] and we have shown that Vpr increases intracellular calcium levels as well as induces NFAT translocation [9]. PHF13 contains two PEST domains which are phosphorylated by the NFAT export kinase GSK3β to regulate PHF13 stability [20]. In addition, it has been suggested that Vpr might regulate the activity of Skp1, the GSK3β homologue in yeast [46] and GSK3β inhibition suppressed Vpr-mediated NFAT translocation [9]. This led us to hypothesize that GSK3β could be involved in Vpr-mediated PHF13 degradation. To analyse this, HIV-1-infected SupT1 cells were treated with the GSK3β inhibitors SB216763 [47] and insulin [48] and PHF13 levels were monitored by immunoblotting (figure 3*c*). Inhibition of GSK3β by both inhibitors stabilized PHF13 and prevented its degradation in HIV-1-infected cells. Hence, enzymes involved in the canonical NFAT pathway, GSK3β and calpains, seem to be involved in Vpr-mediated PHF13 degradation.

To analyse at which stage of the HIV-1 infection cycle PHF13 degradation occurs, we used different inhibitors targeting specific steps of viral replication (figure 3*d*). Western blot analysis revealed that treatment of HIV-1-infected cells with the reverse transcriptase inhibitor Efavirenz and the integrase inhibitor Raltegravir prevented HIV-1-mediated PHF13 degradation. In contrast PHF13 degradation by HIV-1 was unaffected upon treatment with the protease inhibitor Saquinavir and the transcriptional repressor Flavopiridol (figure 3*d*). Altogether, we conclude from these experiments that PHF13 is degraded by HIV-1 Vpr via calpains, the proteasome and GSK3β at a post-integration step, probably before the onset of viral gene expression. In line with this, the integration deficient HIV-1 variant D116N [49] was also impaired in PHF13 degradation (figure 3*e*). These results are also in agreement with our time course experiments (figure 1*b*,*c*), because PHF13 degradation occurs as early as 4 hpi, at a time point when HIV-1 integration is detectable in immortalized T cells [32,50,51].

### 3.4. PHF13 degradation by Vpr mutants and primary lentiviral Vpr alleles

**Figure 4.**
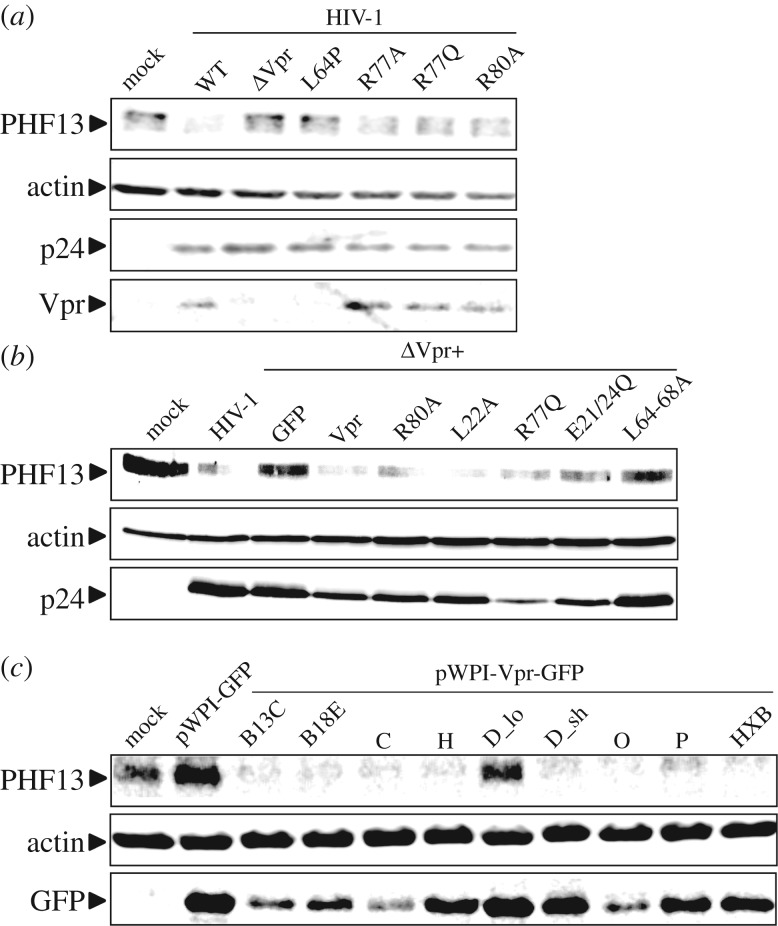
PHF13 degradation by HIV-1 Vpr mutants and primary HIV-1 Vpr alleles. (*a*) SupT1 cells were infected with 200 ng p24 of VSVG pseudotyped HIV-1 NL4-3 IRES-eGFP, the ΔVpr variant or variants carrying the indicated amino acid substitutions in Vpr. Forty-eight hours later, cells were lysed and subjected to immunoblot for detection of PHF13, HIV-1 p24, actin and Vpr. (*b*) SupT1 cells were infected with 200 ng p24 of VSVG pseudotyped HIV-1 NL4-3 or the ΔVpr variant that were transcomplemented in the 293T producer cells with the indicated Vpr mutant, WT Vpr or GFP only as a control. Forty-eight hours later, cells were lysed and subjected to immunoblot for detection of PHF13, HIV-1 p24 and actin. (*c*) SupT1 cells were infected with 200 ng of bicistronic pWPI-GFP lentiviral reporter constructs co-expressing GFP and the indicated primary HIV-1 Vpr alleles, Vpr from the laboratory-adapted HIV-1 reference strain HXB as a control, or GFP only. Forty-eight hours later, cells were lysed and subjected to immunoblot for detection of PHF13, actin and GFP. The data presented in (*a*)–(*c*) were confirmed in at least two additional biological replicates.

Next, we aimed to extend our characterization of Vpr-mediated PHF13 degradation by the functional analyses of various previously described Vpr mutants [9,27]. We infected SupT1 cells with HIV-1 NL4-3 containing either a disrupted Vpr ORF or known mutations at positions L64, R77 or R80 (figure 4*a*). Vpr L64P is unstable, not incorporated into virions [9] and as expected does not degrade PHF13. The R77A/Q change and the R80A mutations do not disrupt Vpr's ability to degrade PHF13. Notably, R77A/Q and R80A are associated with induction of cell death, whereas only R80A has lost the capacity to arrest cells in G_2_ [12]. We corroborated these results by a transcomplementation approach, in which virus producing 293T cells are transfected to co-express Vpr. Hence, in the newly infected cell, Vpr stems only from incoming virions, but is not produced from integrated proviral DNA (compare [9] and figure 2*c*). Consistent with the data in figures 2*c* and 4*a*, virion-delivered Vpr was sufficient to degrade PHF13 and this was independent of mutations R80A or R77Q (figure 4*b*). Similar to the L64P variant, L64-68A is not incorporated into virions [9] and hence defective in PHF13 degradation. Mutants L22A and E21/24Q do not oligomerize and are impaired in inducing PARP1 translocation [9,12]. Nevertheless, both mutants degraded PHF13 (figure 4*b*), suggesting that Vpr-mediated PHF13 degradation is not coupled to these functions. In sum, the results support our model of PHF13 degradation by incoming Vpr and this phenomenon seems functionally unrelated to Vpr-mediated G_2_ arrest.

Because HIV-1 NL4-3 is a laboratory-adapted strain, we aimed to clarify if primary Vpr alleles are also capable to degrade PHF13 and if this function is conserved among HIV-1 groups. From Eric Cohen we kindly obtained a collection of primary Vprs ligated into the lentiviral pWPI backbone, expressing Vpr and GFP from a bicistronic mRNA via an IRES [30]. Upon infection of SupT1 cells with Vpr-containing (and expressing) VLPs, all primary Vpr alleles, except the HIV-1 M subtype D_lo variant degraded PHF13 with a potency similar to Vpr from the laboratory-adapted HIV-1 HXB, which serves as a reference here (figure 4*c*). This is not surprising, because Vpr D_lo is C-terminally elongated, mislocalizes to the cytoplasm and was also inactive in other Vpr functions [30]. In contrast and in agreement with our inhibitor experiments and mutagenesis approach, HIV-1 Vpr Group P which does not arrest cells in G_2_ [30], markedly reduced PHF13 expression (figure 4*c*). In conclusion, PHF13 degradation is a conserved function of primary HIV-1 Vpr alleles.

### 3.5. PHF13 overexpression prior to infection enhances HIV-1 integration

**Figure 5.**
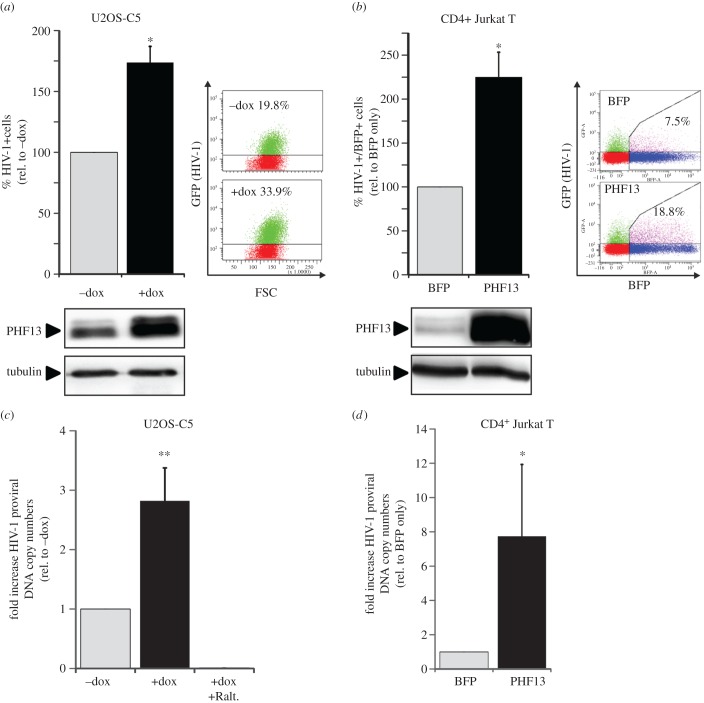
PHF13 expression prior to infection increases the number of integrated proviral genomes and infected cells. (*a*) U2OS-C5 cells were treated with 1 µg ml^−1^ doxycycline or left untreated for 24 h before cells were infected with 100 ng p24 VSVG pseudotyped HIV-1 NL4-3 IRES-eGFP. Twenty-four hours postinfection cells were analysed by flow cytometry. The mean percentage of GFP+ cells from three independent experiments was calculated and the resulting data were normalized to untreated cells (100%). Further shown are representative FACS plots from one experiment and the corresponding immunoblot to control for efficient PHF13 overexpression. (*b*) The same set-up as in (*a*), however Jurkat-TAg cells were microporated with pCG-PHF13-IRES-BFP or BFP-only expression plasmids. Twenty-four hours post-infection cells were analysed by flow cytometry and the mean percentage of PHF13 (BFP+)/HIV-1 infected (GFP+) double-positive cells of three independent experiments was calculated and normalized to the BFP-only control (100%). (*c*,*d*) Genomic DNA of U2OS-C5 and Jurkat-TAg cells treated as described in (*a*) and (*b*) was isolated and analysed for the relative number of integrated proviruses as described in the methods section. Mean fold of relative proviral copies of (*c*) three replicates with two independent virus stocks in U2OS-C5 cells (*n* = 6) and (*d*) two replicates with two independent stocks (*n* = 4) in Jurkat-TAg cells are presented. To control for complete inhibition of integration, doxycycline induced and infected U2OS-C5 cells were also treated with 250 nM Raltegravir (*c*). Error bars indicate the standard deviation. Statistics were calculated with an unpaired two-tailed Student's *t*-test. *p < 0.05; **p < 0.01.

We next aimed to elucidate the biological function of PHF13 within the HIV-1 replication cycle. For this, we used two experimental systems: (i) U2OS-Clone 5 cells in which PHF13 expression can be induced by treatment with doxycycline [20] and (ii) Jurkat-TAg cells transiently electroporated with a pCG-CMV driven reporter construct coexpressing PHF13 and mTagBFP via an IRES. PHF13 overexpression was induced in U2OS-C5 cells and 24 h later cells were infected with HIV-1 NL4-3 GFP, allowing for quantitation of HIV-1 infection by measuring the percentage of GFP-expressing cells. Treatment with doxycycline induced PHF13 overexpression and the number of HIV-1-infected (GFP+) cells increased nearly 1.8-fold (figure 5*a*). This was not observed in the parental cell line U2OS (electronic supplementary material, figure S2). The same was true for CD4^+^ Jurkat cells, which were electroporated to express PHF13 (figure 5*b*), such that in BFP-positive cells, which overexpressed PHF13, HIV-1 infection was roughly twofold more efficient when compared with Jurkat cells electroporated with a BFP-only control plasmid (figure 5*b*). Hence, PHF13 appeared to enhance the number of infected cells when it was expressed prior to infection of target cells, although PHF13 was suggested to act as HIV-1 restriction factor [23].

PHF13 is involved in the regulation of DNA repair [17,20] and chromatin-associated through direct binding to H3K4me2/3 [21], which is superimposed on HIV recurrent integration genes [52]. This prompted us to test the effect of PHF13 on the number of integrated proviral genomes. Samples from PHF13 overexpressing and HIV-1-infected U2OS-C5 and Jurkat cells were taken at 24 hpi, and genomic DNA was extracted to quantify the number of integrated proviruses by Alu-PCR (figure 5*c*,*d*). Cells treated with the integration inhibitor Raltegravir (+Ralt.) during infection served as negative control for the absence of proviral integration. This analysis revealed that PHF13 overexpression prior to HIV-1 infection leads to a higher number of integrated proviral genomes; in U2OS-C5 cells up to threefold and in Jurkat T cells with approximately eightfold increased efficiency.

### 3.6. PHF13 overexpression suppresses HIV-1 gene expression at the post integration stage

**Figure 6.**
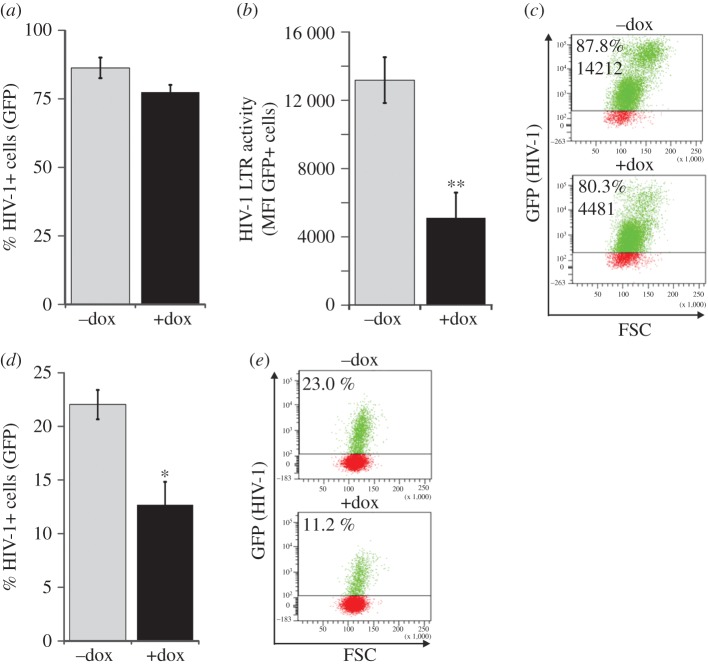
PHF13 expression post-infection suppresses HIV-1 gene expression and production of infectious virions. U2OS-C5 cells were infected with 100 ng p24 VSVG pseudotyped HIV-1 NL4-3 IRES-eGFP for 24 h. Thereafter, cells were treated with 1 µg ml^−1^ doxycycline to induce PHF13 expression. Additional 24 h later cells were analysed by flow cytometry. (*a*) Mean percentage of HIV-1 infected (GFP+) cells. (*b*) Mean fluorescence intensity (LTR transactivation) of the GFP+ cells. (*c*) Representative FACS plots of the data presented in (*a*) and (*b*). The percentage of infected cells and the GFP MFI is indicated. (*d*) Supernatants from the U2OS-C5 cells harvested at 48 hpi were used to inoculate SupT1 cells. Twenty-four hours later SupT1 were analysed by flow cytometry for HIV-1 infection (% GFP+ cells), which is a marker for the production of infectious virions from the U2OS-C5 cells which were treated as described in (*a*) and (*b*). (*e*) Representative FACS plots from the experiment described in (*d*). Mean values and standard deviations were calculated from six (*a* and *b*) and three (*d*) independent infections. Statistics were done with an unpaired two tailed Student's *t*-test. *p < 0.05; **p < 0.01.

PHF13 appears to increase HIV-1 proviral copy numbers, but is then degraded. This implies that PHF13 expression could have antiviral effects at the post integration stage of the HIV-1 replication cycle. In order to test this hypothesis, PHF13-inducible U2OS-C5 cells were infected with HIV-1 NL4-3 GFP. Twenty-four hours later PHF13 overexpression was induced by addition of doxycycline to the cell culture media followed by FACS analysis 48 hpi. In contrast to what we observed when PHF13 was overexpressed prior to infection (figure 5), the total number of HIV-1-infected cells (% GFP+) remained comparable between doxycycline induced and non-induced cells (figure 6*a*). However remarkably, the GFP mean fluorescence intensity, which is a marker for HIV-1 gene expression, was reduced in PHF13 overexpressing U2OS-C5 cells (figure 6*b*,*c*). This phenotype was clearly PHF13 dependent because it was absent in infected but mock-treated or parental U2OS cells (electronic supplementary material, figure S3). Furthermore, supernatants from doxycycline induced and HIV-1-infected U2OS-C5 cells contained approximately half the amount of infectious HIV-1 particles in comparison to the controls (figure 6*d*,*e*). In conclusion, PHF13 inhibits HIV-1 gene expression and production of progeny virions in the post-integration phase of the viral replication cycle.

### 3.7. HIV-1 Vpr counteracts PHF13-mediated inhibition of viral gene expression

**Figure 7.**
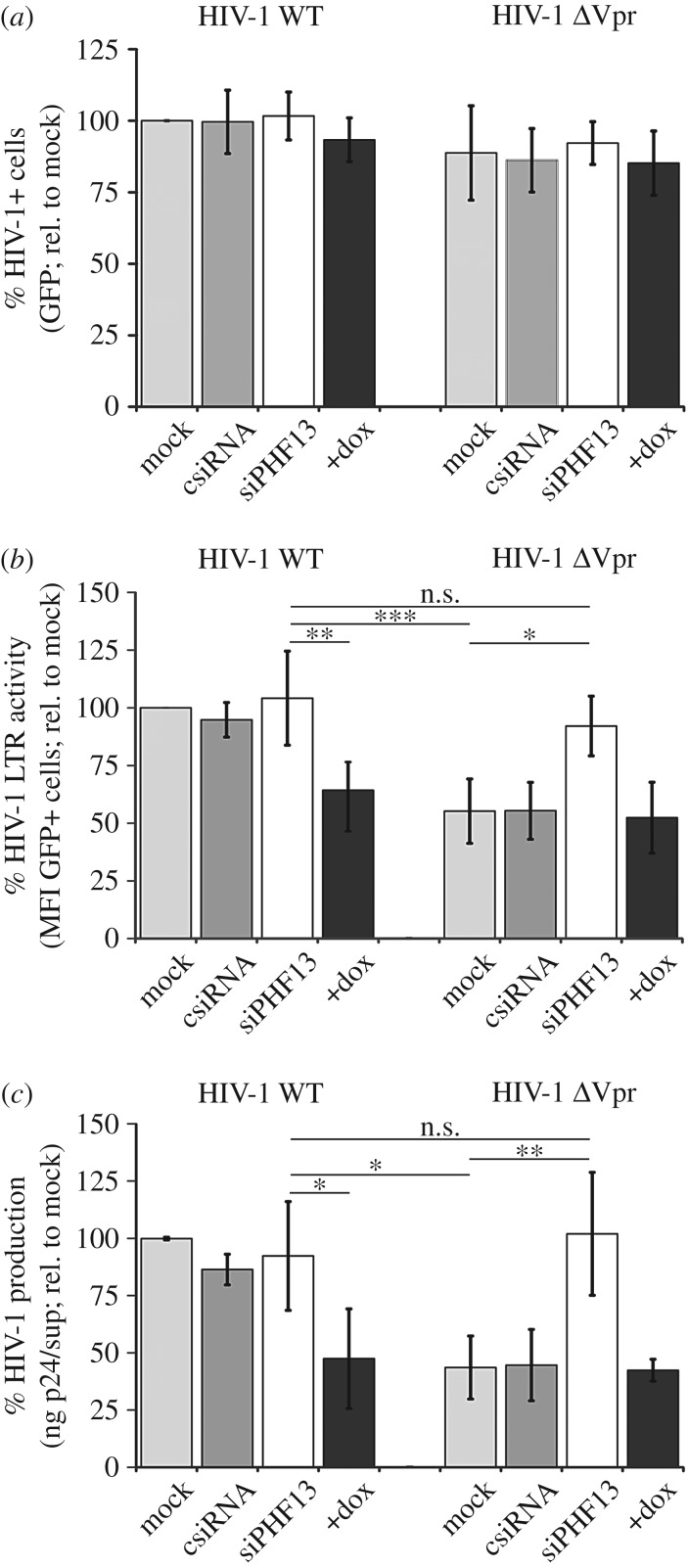
Vpr counteracts PHF13-mediated suppression of viral gene expression. U2OS-C5 cells were either mock transfected or transfected with control siRNA (csiRNA) or siRNA constructs directed against PHF13 (siPHF13). Twenty-four hours later cells were infected with 100 ng p24 VSVG pseudotyped HIV-1 NL4-3 IRES-eGFP or the ΔVpr variant for 6 h, washed and incubated for additional 18 h in medium with or without 1 µg ml^−1^ doxycycline. Twenty-four hours later cells were analysed by flow cytometry or ELISA for (*a*) the percentage of GFP+, hence HIV-1-infected cells, (*b*) MFI of the GFP+ cells as marker for LTR transactivation and (*c*) the amount of p24 released in the supernatants. Mean values and standard deviations were calculated from four (*a* and *b*) or three (*c*) independent experiments and normalized to the HIV-1-infected and mock-treated control. Statistics were calculated with one-way analysis of variance (ANOVA) with a Bonferroni multiple comparison post-test. *p < 0.05; **p < 0.01; ***p < 0.001; n.s., not significant.

Inhibition of viral gene expression imposed by PHF13 could be antagonized by Vpr. To challenge this hypothesis, PHF13 inducible U2OS-C5 cells were infected with equal amounts of WT HIV-1 or the ΔVpr mutant. Simultaneously, PHF13 expression was suppressed by siRNA knock-down or induced by treatment with doxycycline. 48 hpi cells and supernatants were harvested and analysed by FACS and p24 ELISA (figure 7). As expected, when PHF13 is overexpressed or knocked down at the post-integration step, the total percentage of HIV-1-infected (% GFP+) cells was comparable between all infections (figure 7*a*). Furthermore, cells were lysed and subjected to immunoblotting. This confirmed equal infection efficiencies by p24 detection, the efficiency of siRNA-mediated PHF13 knock-down and PHF13 overexpression when induced with doxycycline, as well as PHF13 degradation by Vpr (electronic supplementary material, figure S4). Analysis of GFP mean fluorescence intensity as an indirect marker for HIV-1 LTR activity and viral gene expression showed no influence of PHF13 knockdown when cells were infected with HIV-1 WT (figure 7*b*). As PHF13 is efficiently degraded by Vpr, this phenotype was expected. Of note, when PHF13 was doxycycline induced in HIV-1 WT-infected cells viral gene expression was reduced to the level of mock or control siRNA transfected cells infected with ΔVpr HIV-1. Conversely, knockdown of PHF13 in ΔVpr HIV-1 infections led to a complete recovery of viral gene expression similar to HIV-1 WT (figure 7*b*).

As an independent readout for viral gene expression and production of progeny virions we took supernatants of the same cells and measured the amount of released HIV-1 p24 capsid (figure 7*c*). This analysis mirrored the effects that we observed previously when using GFP mean fluorescence intensity of infected cells as an indicator for the efficiency of viral gene expression. These experiments demonstrate that PHF13 interferes with HIV-1 gene expression and its activity is counteracted by Vpr.

## 4. Discussion

**Figure 8.**
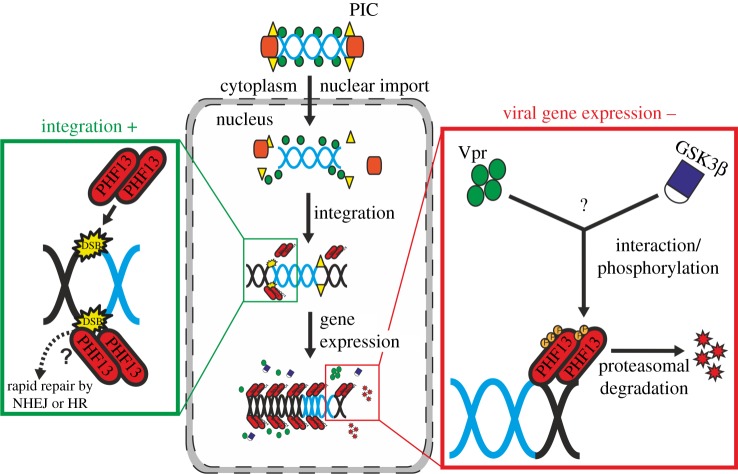
Model of the early and post-integration effects of PHF13 during the HIV-1 replication cycle. After entry into the nucleus the HIV-1 DNA genome is integrated into the host genome. As a consequence, the host cell DNA suffers a DSB. PHF13 is recruited to DSBs, could regulate the DNA DSB repair machinery and positively influence HIV-1 integration possibly by linking nuclear non-integrated HIV-1 DNA to H3K4me2/3 actively transcribed regions at the nuclear periphery. After integration PHF13 might be degraded through the concerted action of HIV-1 Vpr, GSK3β, calpain and the proteasome to counteract PHF13 imposed inhibition of HIV-1 gene expression.

In this study, the role of the nuclear protein and putative restriction factor PHF13 for HIV-1 replication was investigated. PHF13 was shown to increase proviral integration and hence the total number of infected cells. Nonetheless, PHF13 was degraded by the viral accessory protein Vpr after integration (figure 8). This degradation probably evolved due to inhibitory effects of PHF13 on viral gene expression. Hence, PHF13 seems initially important for efficient HIV-1 integration. At later steps, Vpr degrades PHF13 to counteract its antiviral functions.

### 4.1. Vpr induces PHF13 degradation independent of its interaction with the CUL4 E3 ubiquitin ligase complex

Vpr is a multi-functional HIV-1 accessory protein that is assumed to play an important role during the early phase of infection. This includes increased nuclear import of the viral pre-integration complex (PIC), enhancement of HIV-1 reverse transcription and induction of the G_2_ cell cycle arrest [7]. Interestingly, Vpr can cause epigenetic disruption of heterochromatin by inducing displacement of heterochromatin protein 1-α (HP1-α) through acetylation of histone H3 [53]. As HP1-α is in a complex with PHF13 [17,20], Vpr-mediated PHF13 degradation could be mechanistically linked to this phenotype. Moreover, other proteins involved in DNA repair (i.e. uracil-DNA glycosylase 2, single-strand selective monofunctional uracil-DNA glycosylase and more recently the DNA helicase/translocase HLTF) are degraded by Vpr through an E3 ubiquitin ligase complex composed of VPRBP (Vpr binding protein or DCAF), DDB1 and cullin 4A (CUL4A) [38–40,44,54]. Using the neddylation and CUL4A inhibitor MLN4924 we still observed efficient PHF13 degradation by Vpr. Furthermore, Vpr mutants and the primary HIV-1 P variant, which do not associate with CUL4A or cause G_2_ arrest (figure 4) [30], efficiently degraded PHF13. Accordingly, the Vpr-associated CUL4A ubiquitin ligase seems inessential for PHF13 degradation. In contrast, our data suggest that Vpr mediates PHF13 depletion through the proteasome involving GSK3β and calpains. Consistent with our findings, PHF13 stability is regulated by GSK3β [20] and Vpr has been proposed to influence the activity of the GSK3β homologue in yeast Skp1 [46]. Accordingly, we recently reported that virion-delivered Vpr enhances intracellular calcium and induces NFAT translocation [9], supporting the notion that Vpr has the capability to dysregulate cellular players in the canonical NFAT pathway (i.e. GSK3β and calpains).

The exact mechanism of Vpr-mediated PHF13 degradation remains to be established. GSK3β substrates (i.e. PHF13) need to be pre-phosphorylated by a priming kinase before GSK3β can bind and hyperphosphorylate them [55]. Hence, it is tempting to speculate that Vpr directly or indirectly pre-phosphorylates PHF13 by hijacking an unknown intermediate player. Intriguingly, Vpr can activate Wee1 kinase, which subsequently leads to phosphorylation of cellular targets, for instance p34-cdc2 [56]. Since we were not able to show a direct interaction between Vpr and PHF13, or Vpr and GSK3β, by FRET and coimmunoprecipitation experiments, (data not shown) we argue in favour of the latter. The timely activation of such a nuclear kinase by Vpr seems realistic, considering the rapid transport of virion-borne Vpr into the nucleus as recently elegantly shown by sophisticated imaging techniques [57], which is also in line with our observation of rapid PHF13 degradation as early as 4 h after infection.

### 4.2. Mechanistic explanations for the enhancement of HIV-1 integration by PHF13

HIV-1 integration induces DNA DSBs of the genome and the two major pathways for DSB repair in mammalian cells, the non-homologous end joining (NHEJ) or homologous recombination repair (HR) are activated [58]. Further, PHF13 depletion enhances NHEJ repair activity but impairs HR, and conversely, overexpression of PHF13 reduces NHEJ repair activity [17]. In our experiments high levels of PHF13 enhanced HIV-1 integration, arguing that HR could be more important for viral integration than NHEJ.

The TRIM family protein KAP-1 inhibits HIV-1 integration by binding to acetylated integrase and inducing its deacetylation, thereby negatively regulating integrase activity [59]. KAP-1, similar to PHF13, is also antiviral against adenoviruses [60]. As PHF13 interacts with KAP-1 and modulates its chromatin association [17] it could sequester KAP-1 and interfere with KAP-1-mediated inhibition of HIV-1 integration. Another aspect is that HIV-1 preferentially integrates in chromatin localized at the periphery of the nucleus, at the borders of heterochromatic Lamin-associated domains (LADs) [52,61,62]. Notably, H3K4me2/3 is superimposed on HIV recurrent integration genes at LAD borders [52], and recently Chung *et al.* [21] demonstrated by a series of experiments direct binding of PHF13 to H3K4me2/3. In conclusion, PHF13 could direct non-integrated HIV-1 DNA to these active sites of heterochromatin at the nuclear periphery. Altogether, the different functions associated with PHF13 are in line with our experimental findings. In the future, it will be highly interesting to delineate which feature(s) of PHF13 are associated with enhanced HIV-1 integration, if and how there is an interplay with the main HIV-1 integration factor LEDGF [63], and how PHF13 influences HIV-1 nuclear distribution.

### 4.3. PHF13-mediated restriction of HIV-1 gene expression is antagonized by Vpr

After integration, PHF13 leads to a reduction of HIV-1 gene expression and virus production and release. PHF13 transforms chromatin into a more condensed heterochromatin form [20], which could be associated with suppression of gene expression. For proper transcriptional activation of the viral genome, DNA has to be in a decondensed form (euchromatin) to allow access of transcription factors and Tat [64,65]. Hence, one theory is that PHF13 represses viral gene expression at the level of transcription by formation of heterochromatin.

The expression level and localization of PHF13 are dynamically regulated during the cell cycle and might play a role during cell division [20]. In early G_1_–S, as well as early S phase, PHF13 expression levels are significantly decreased but during late G_2_ and M phases PHF13 levels are increased. This indicates that PHF13 might be important during late G_2_ and could drive cell cycle progression. In the G_2_ phase the HIV-1 LTR is in a higher transcriptional activation state and it was postulated that Vpr induces G_2_ arrest to promote efficient LTR transactivation [7,66–68]. Hence, it can be speculated that Vpr might degrade PHF13 to prevent cell cycle transition from G_2_ to G_1_. While this is an attractive hypothesis, our data argue against PHF13 as essential factor for the Vpr induced G_2_ arrest: (i) PHF13 knockdown in U2OS cells did not promote G_2_ cell cycle arrest (data not shown), (ii) the neddylation and CUL4A ubiquitin ligase inhibitor MLN4924 did not block Vpr-mediated PHF13 degradation, and Vpr mutants and variants that were inactive in inducing G_2_ arrest efficiently degrade PHF13. The ability of Vpr to induce G_2_ arrest correlates with its association with the CUL4A ligase complex [13,69]. Therefore, in line with our data, both activities are probably separable and functionally independent.

PHF13 knockdown in ΔVpr infections led to levels of HIV-1 gene expression and virus release which were comparable to the levels observed with HIV-1 WT. While the enhancing effect of Vpr on HIV-1 LTR transactivation has been reported several times, the mechanistic explanation for this phenotype was lacking [7]. With PHF13, we here identify the cellular factor interfering with HIV-1 gene expression. Clearly, this factor is degraded and antagonized by Vpr.

PHF13 directly binds to H3K4me2/3 and is a transcriptional co-regulator, resulting in the up- and downregulation of a multitude of genes involved in transcriptional regulation, chromatin reorganization, cell cycle and differentiation [21]. Its activity on transcription is probably due to its formation of a complex with the RNA PolII and the PRC2. Indeed, Chung *et al.* [21] have nicely shown that PHF13 depletion disrupted this complex and leads to increased expression of genes with high H3K4me2, H3K27me3 and other histone marks. Notably, early LTR transcription is silenced by PRC2 and H3K27me3, which also leads to HIV-1 dormancy [70]. We favour and put forward a model in which PHF13 in complex with PRC2 silences HIV-1 gene expression at H3K4me2/3 integration sites. This block is alleviated by Vpr-mediated PHF13 depletion. Of note, the multitude of Vpr effects on several cellular processes [7,12,71] could be explained by the depletion of PHF13 and its emerging role in transcriptional regulation and co-transcriptional splicing [22].

Counteraction of cellular restriction factors is probably the most important feature of lentiviral accessory proteins. Vif antagonizes the APOBEC3G deaminase [72–75], Vpu and Nef counteract Tetherin [35,36,76,77], and Nef counteracts SERINC3/5 to maintain infectivity of viral particles [78–80]. Vpx, which is expressed by HIV-2 and some SIVs, neutralizes the antiviral activity of the nucleotide hydrolase SAMHD1 [81,82]. Other antiviral host cell proteins (e.g. Trim5α) have been evaded by the evolution of HIV-1 capsids which are not sensed by this factor [83,84]. It is noteworthy that all these cellular restriction pathways act at different stages of the viral replication cycle. Trim5α prevents uncoating of the capsid, APOBEC3G hypermutates the viral genome, SAMHD1 interferes with reverse transcription, Tetherin with virus release and SERINC3/5 with HIV-1 infectivity. With PHF13 we now add a factor interfering with HIV-1 gene expression. As PHF13 also dampens adenoviral gene expression [23], it is likely that, similar to the restriction factors discussed above, it could be part of the innate antiviral host defense machinery acting against a broad panel of viruses. However, it has to be noted that PHF13, similar to SERINC3/5, is not interferon-α induced (electronic supplementary material, figure S5) [78,79].

Studies answering the above questions, as well as investigation of the effects of Vpx and simian immunodeficiency virus, and primary patient-derived HIV-1 Vpr alleles on PHF13 expression, are of high relevance.

### 4.4. Potential role of Vpr-mediated PHF13 degradation in primary HIV-1 target cells, macrophages and CD4^+^ T cells

PHF13 is expressed in most cell lines and primary cells, albeit we observe substantially lower levels of PHF13 in primary macrophages in comparison to CD4^+^ T cells (figure 1*a*). As PHF13 is a transcriptional regulator and active in cycling cells, this could explain the low abundance in macrophages. Of note, recent data demonstrate that HIV-1 preferentially infects G1-like cycling macrophages, expressing phosphorylated and thus antiviral inactive SAMHD1 [85]. If this minor population of macrophages expresses high levels of PHF13 which could be degraded by Vpr remains to be addressed. However, such a scenario could explain the importance of Vpr for viral replication in macrophages [27,86–89]. On the other hand, PHF13 is robustly degraded in HIV-1-infected CD4^+^ T cells by Vpr. Also in certain immortalized T cell lines HIV-1 Vpr was shown to have a positive effect on viral replication [11,68]. It further clearly enhances replication in human lymphoid tissue, mainly containing CD4^+^ T cells [8,27], and we could recently demonstrate Vpr-mediated enhancement of non-stimulated CD4^+^ T cell infection [9]. Hence, the aforementioned effects of Vpr and concomitant degradation of PHF13 are probably relevant in primary HIV-1 target cells. Furthermore, by favouring HIV-1 genome integration and repressing viral transcription PHF13 might act as a latency promoting factor. To clarify this highly relevant question further experimentation is required and warranted.

## 5. Conclusion

The results of this study suggest that PHF13 has opposing effects throughout the HIV-1 replication cycle (figure 8). After viral entry and nuclear import of the PIC, PHF13 can increase the number of integrated HIV-1 proviral genomes. After integration, PHF13 acts as antiviral restriction factor and inhibits viral gene expression. HIV-1 counteracts this suppressive effect on gene expression by Vpr, which promotes degradation of PHF13. Nevertheless, due to its positive effects on integration PHF13 is not a bona fide restriction factor. Fascinatingly, HIV-1 appears to have evolved a sophisticated and highly regulated mechanism to exploit PHF13 for the optimization of viral replication and virus production.

## Supplementary Material

Figure S1: HIV-1 reduces PHF13 expression in SupT1 and Jurkat-TAg cells

## Supplementary Material

Figure S2: No effects of doxycycline treatment on HIV-1 infection in parental U2OS cells.

## Supplementary Material

Figure S3: No effects on HIV-1 LTRtransactivation when parental U2OS cells were treated with doxycycline post infection

## Supplementary Material

Figure S4: Analysis of knockdown efficiency in HIV-1-infected U2OS-C5 cells

## Supplementary Material

Figure S5: Effect of Interferon-α on PHF13 expression

## Data accessibility

This article has no additional data.
