# Supplementary material for: Dual role of the chromatin-binding factor PHF13 in the pre- and post-integration phases of HIV-1 replication
Source: Open Biol. 2017 Oct 11;7(10):170115. doi: 10.1098/rsob.170115 (PMC5666080; doi:10.1098/rsob.170115)
Supplement: Figure S1: HIV-1 reduces PHF13 expression in SupT1 and Jurkat-TAg cells [file rsob170115supp1.pdf]

Hofmann et al., Fig.S1

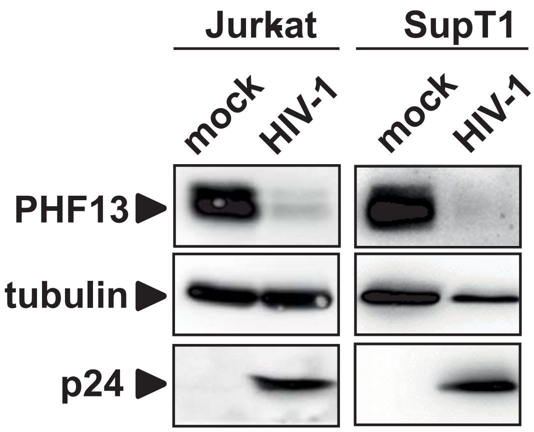

Figure S1: HIV-1 reduces PHF13 expression in SupT1 and Jurkat-TAg cells. Jurkat-TAg or SupT1 cells were infected with 200 ng p24 VSVG pseudotyped HIV-1 NL4-3 or mock infected. Cells were harvested 48 hpi, total cell extracts were prepared and analyzed for expression of PHF13, HIV-1 capsid p24 and tubulin by immunoblot.
