# Supplementary material for: Dual role of the chromatin-binding factor PHF13 in the pre- and post-integration phases of HIV-1 replication
Source: Open Biol. 2017 Oct 11;7(10):170115. doi: 10.1098/rsob.170115 (PMC5666080; doi:10.1098/rsob.170115)
Supplement: Figure S2: No effects of doxycycline treatment on HIV-1 infection in parental U2OS cells. [file rsob170115supp2.pdf]

Hofmann et al., Fig.S2

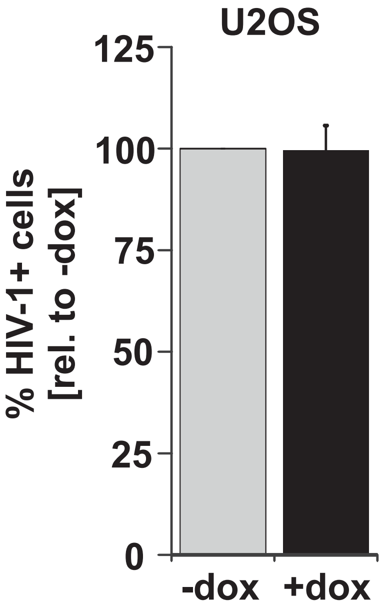

Figure S2: No effects of doxycycline treatment on HIV-1 infection in parental U2OS cells. U2OS cells were treated with 1 µg/ml doxycycline or left untreated for 24 h before cells were infected with 100 ng p24 VSVG pseudotyped HIV-1 NL4-3 IRES-eGFP. 24 hpi cells were analyzed by flow cytometry. The mean percentage of GFP+ cells from three independent experiments was calculated and the resulting data was normalized to untreated cells (100 %).
