# Supplementary material for: Dual role of the chromatin-binding factor PHF13 in the pre- and post-integration phases of HIV-1 replication
Source: Open Biol. 2017 Oct 11;7(10):170115. doi: 10.1098/rsob.170115 (PMC5666080; doi:10.1098/rsob.170115)
Supplement: Figure S3: No effects on HIV-1 LTRtransactivation when parental U2OS cells were treated with doxycycline post infection [file rsob170115supp3.pdf]

Hofmann et al., Fig.S3

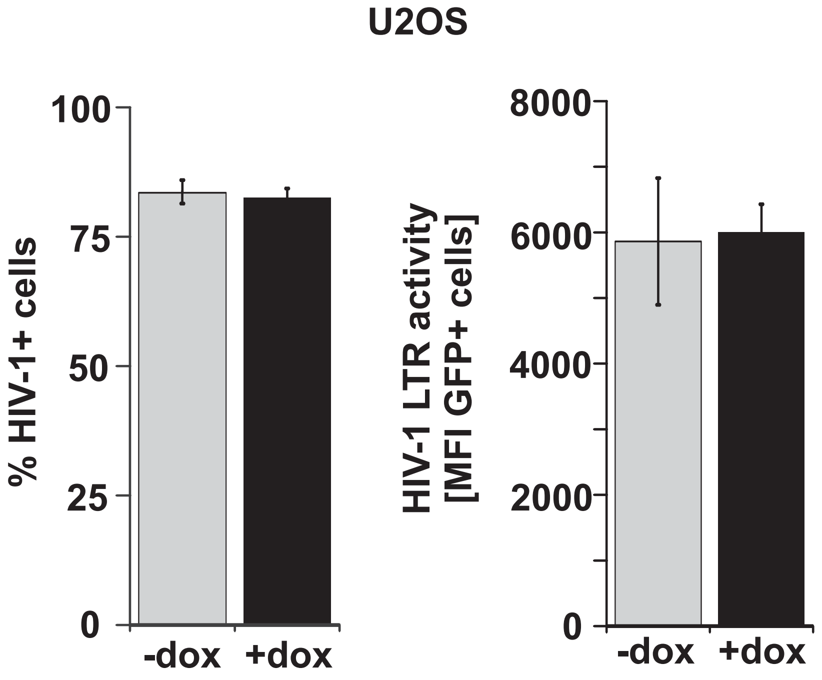

Figure S3: No effects on HIV-1 LTR transactivation when parental U2OS cells were treated with doxycycline post infection. U2OS cells were infected with 100 ng p24 VSVG pseudotyped HIV-1 NL4-3 IRES-eGFP for 24 h. Thereafter, cells were treated with 1 µg/ml doxycycline. Additional 24 h later cells were analyzed by flow cytometry for the % and MFI of GFP+ (HIV-1-infected) cells. The graph shows mean values and standard deviations of three independent experiments.
