# Supplementary material for: Dual role of the chromatin-binding factor PHF13 in the pre- and post-integration phases of HIV-1 replication
Source: Open Biol. 2017 Oct 11;7(10):170115. doi: 10.1098/rsob.170115 (PMC5666080; doi:10.1098/rsob.170115)
Supplement: Figure S4: Analysis of knockdown efficiency in HIV-1-infected U2OS-C5 cells [file rsob170115supp4.pdf]

Hofmann et al., Fig.S4

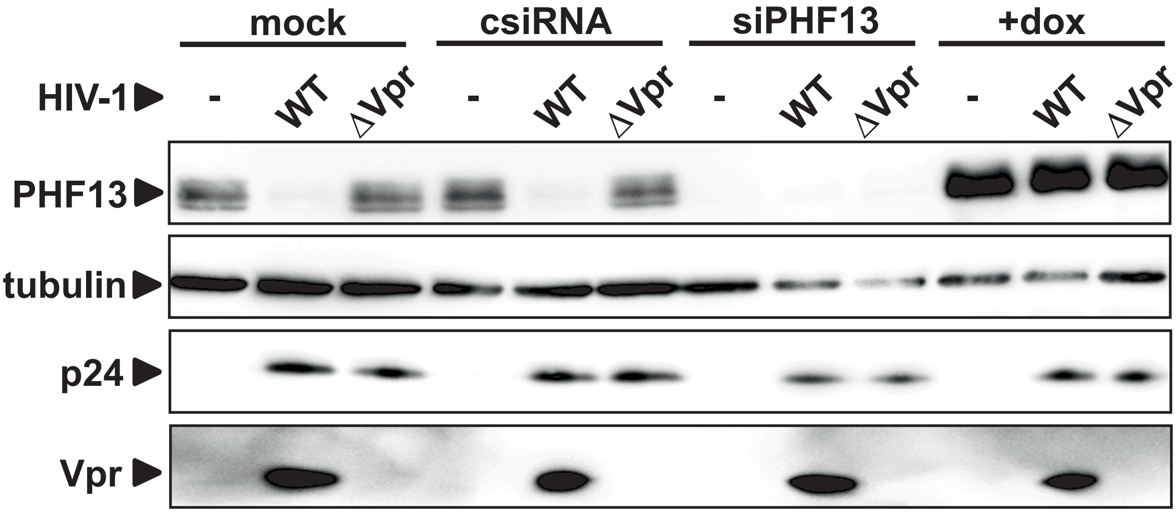

Figure S4: Analysis of knockdown efficiency in HIV-1-infected U2OS-C5 cells. U2OS-C5 cells were mock transfected or with a control siRNA (csiRNA) or directed against PHF13 (siPHF13). 24 h later cells were infected with 100 ng p24 VSVG pseudotyped HIV-1 NL4-3 IRES-eGFP or the ΔVpr variant for 6 h, washed and incubated for additional 18 h. At that time point, cells were induced with 1 µg/ml doxycycline. 24 h later cells were lysed to detect PHF13, tubulin, HIV-1 p24 and Vpr by immunoblot.
