# Supplementary material for: Dual role of the chromatin-binding factor PHF13 in the pre- and post-integration phases of HIV-1 replication
Source: Open Biol. 2017 Oct 11;7(10):170115. doi: 10.1098/rsob.170115 (PMC5666080; doi:10.1098/rsob.170115)
Supplement: Figure S5: Effect of Interferon-α on PHF13 expression [file rsob170115supp5.pdf]

Hofmann et al., Fig.S5

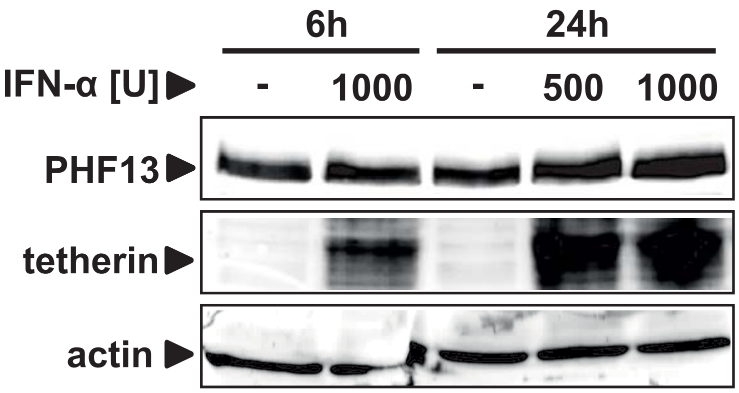

Figure S5: Effect of Interferon-α on PHF13 expression. 293T cells were treated with the indicated amounts of recombinant Interferon-**α** and cultured for 6 h or 24 h. At the time points indicated cells were lysed to detect PHF13, tetherin and actin by immunoblot.
